# Assessing wineries' performance in managing critical control points for arsenic, lead, and cadmium contamination risk in the wine-making industry: A survey-based analysis utilizing performance indicators as a results tool

**DOI:** 10.1016/j.heliyon.2023.e22962

**Published:** 2023-12-05

**Authors:** Jesús López-Santiago, Ana Isabel García García, Alejandro Gómez Villarino, Amelia Md Som, María Teresa Gómez-Villarino

**Affiliations:** a Agroforestry Engineering Department, School of Agricultural, Food and Biosystems Engineering, Universidad Politécnica de Madrid, Spain; b Independent Consultant, Spain; c Malaysian Institute of Chemical & Bioengineering Technology (UniKL MICET), Universiti Kuala Lumpur, Malaysia

**Keywords:** Food safety, Food hazards, HACCP, Critical control points, Beverages

## Abstract

Human health hazards appear in wine production. Wineries have implemented food safety management systems to control food hazards through Hazard Analysis Critical Control Point (HACCP). Wine-making industry applies HACCP by evaluating Critical Control Points (CCPs). One of the CCPs that exhibits inadequate control is the potential contamination risk of arsenic, cadmium, and lead throughout the winemaking procedure. Wineries performance level about controlling CCPs related to contamination risk by arsenic, cadmium and lead in the winemaking were analyzed. A sixteen-question questionnaire was made to achieve this research. Three indicators were calculated for training, legislation, and analysis performance components in CCPs control. Results revealed that wineries fault in analysis and legislation components. Identification and updating of legislation about As, Cd and Pb contamination risk is in starting performance level for wineries that produce less than 250,000 L/year wineries. Analysis performance level is even lower than legislation. Only one out of every three wineries possess information regarding the concentrations of arsenic, cadmium, and lead in the soils of vineyards where grapes are cultivated. Furthermore, the availability of data on their available concentrations in the soil solution is even more limited. Those wineries that controlled As, Cd and Pb concentrations make it according to official recommendations using techniques based on atomic absorption spectrometry. However, there is a lack of this spectrometry equipment in the wineries own laboratories.

## 1 Introduction

Food hazards in winemaking arise from various sources, including improper practices by winery staff, equipment and infrastructure used in winemaking, and environmental factors. Cross-contamination and allergens have been identified as the primary causes of food safety incident [1]. The accumulation of residues, which can have physical, chemical, or microbiological origins, is a major concern in wine production. In this context, studies have shown that excessive intake of ions such as arsenic, lead and cadmium can be toxics for human health [2,3].

Metals and metalloids present in grapes primarily come from the soil and the application of fertilizers, pesticides, and fungicides containing substances like cadmium, copper, manganese, lead, or zinc [4]. Spanish soils naturally have elevated concentrations of these elements, particularly arsenic [[5], [6], [7]]. High arsenic concentrations in vineyard soils in central Spain have been reported by Jimenez-Ballesta (2023) [8]. Andersson and Nilsson found that chemical elements like cadmium, lead and arsenic from sewage sludge fertilization (84 t/ha) remained in the top 20 cm of the soil for twelve years [9]. However, vines and grapes are not hyperaccumulators of potentially toxic soil elements (PTEs), with higher concentrations of PTEs found on the outer parts (leaf and petiole) compared to the inner parts of the grape (skin, pulp, and seed) [10].

Controlling metals and metalloids food hazards is achieved by addressing associated food risks. Wineries utilize Food Safety Management Systems (FSMS) as to manage these risks. Good manufacturing practices (GMP) in winery operations establish hygienic conditions aimed at preventing the presence of hazardous agents [11].

FSMS typically include Prerequisites Programmes (PRPs) [12] and a Hazard Analysis Critical Control Point (HACCP) [13,14] in accordance with the reference regulatory framework in the European Union [15]. PRPs ensure appropriated environmental and operational conditions necessary for producing safe and healthy food. PRPs address issues to the supply and use of sanitary water, equipment and facility cleaning and disinfection, pest control and prevention, good manufacturing practices or staff knowledge of food safety, allergens and food traceability [16].

HACCP is a globally recognized and standardized methodology for ensuring food safety. It is based on seven principles that focus on identifying and controlling food safety hazards. The first three principles involve hazard identification, determining CCPs and establishing of critical limits of these CCPs to ensure food safety [17,18]. Also, a HACCP plan encompasses control measures that can be employed to proactively prevent, mitigate, or eliminate potential hazards. Regular monitoring and verification procedures, including rigorous testing, serve to validate the efficacy of CCPs, with swift corrective actions promptly implemented upon detecting any contamination. Meticulous documentation and comprehensive record-keeping throughout the entire HACCP process stand as imperative requirements for ensuring adherence to regulatory standards and facilitating traceability.

A critical control point (CCP) is a point in a step or procedure at which a control must be applied and is essential to prevent or eliminate a food safety hazard or reduce it to an acceptable level [1]. Conducting a hazard analysis of CCPs enables the identification, evaluation, and control of significant CCPs throughout the food production process. Once potential hazards are identified, their status as a CCP is assessed, and reference limits or critical limits are established through the implementation of preventive measures to prevent deviations from these limits [13,16,19]. Identification of CCPs is executed by employing the decision tree framework as outlined by Codex Alimentarius, which was further tailored with ISO 22000: 2018 (E) criteria [20]. This procedure helps determine whether the hazards can be effectively addressed as Operational Prerequisite Programme (oPRPs) or whether specific operational protocols, including defined measures, are required for the management of CCPs [20,21]. Inadequate control of CCPs can lead to contamination of grapes and wines with microorganisms, residues from phytosanitary products, or traces of heavy metal or metalloids from soils.

Christaki, T. (2002) identified main CCPs in red wine production [22]. Benito, S. (2019) conducted a study on the identification and control of CCPs during winemaking to mitigate the levels of various compounds, including biogenic amines, ethyl carbamate, ochratoxin A, and sulfur dioxide [23]. Martinez-Rodriguez (2009) studied CCPs associated with microbial safety during wine production, with a particular focus on Ochratoxin A [24].

**Fig. 1:**
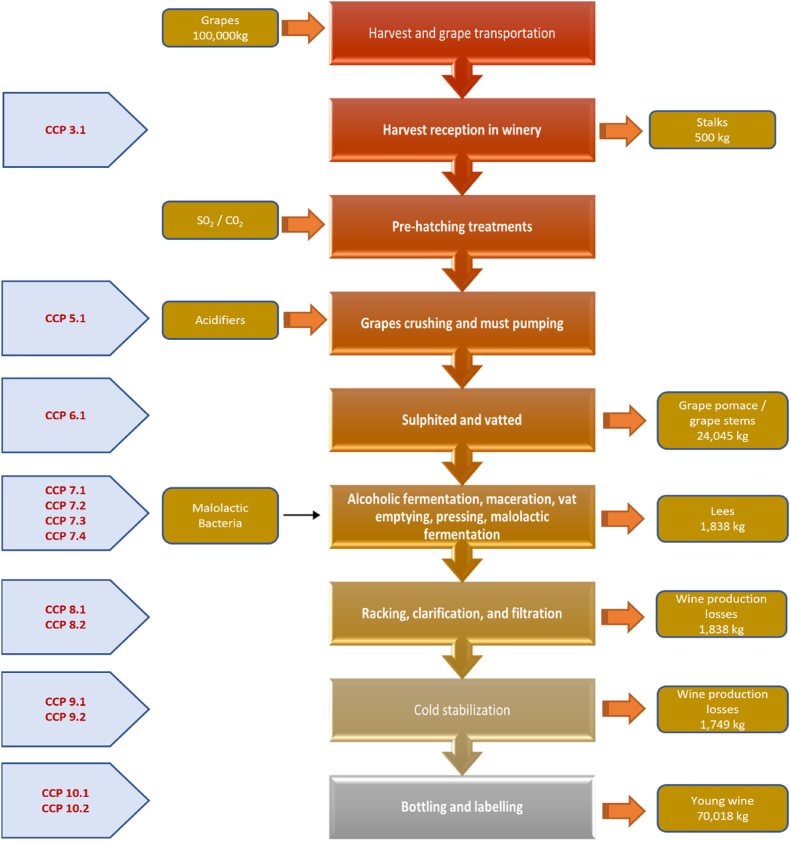
Flow diagram of the young wine making process and corresponding CCPs to each stage of this process.

Table 1 shows main CCPs and oPRPs in the production of young wine based on previous research [20,[22], [23], [24], [25]]. Fig. 1 shows a flow diagram of wine-making process and corresponding CCPs to each stage of this process based on technical document [26] and Table 1.

**Table 1:** Main CCPs and oPRPs in the production of young wine.

Wine-making process steps	Critical Control Point vs Operational Prerequisite Programme
Harvest and grape transportation	oPRP 2.1 Vineyards inspection prior to the harvest to know the general condition of the grapes.
	oPRP 2.2 Vineyards inspection during the harvest to know the state of grapes.
	oPRP 2.3 Time control that takes to transport the harvest to the winery.
Harvest reception in winery	oPRP 3.1 Control of residues of fungicides and/or pesticides existing in grapes intended for winemaking.
	oPRP 3.2 Mycotoxins control from grape rot.
	oPRP 3.3 Control of the presence of contamination by plant debris, dust and/or metallic elements.
	CCP 3.1 Control of the presence of contamination by metals (Cd, Pb, As) in the grapes.
Pre-hatching treatments	oPRP 4.1 Control of the cleanliness of the tanks to eliminate residues of microorganisms.
	oPRP 4.2 Control of the absence of cleaning and disinfection products in the tanks.
Grape crushing and must pumping	oPRP 5.1 Control of the cleanliness of crushing equipment.
	oPRP 5.2 Control of the absence of cleaning and disinfection products in tanks, press and pumping equipment.
	CCP 5.1 Control of the wort maintenance time in the crusher.
Sulphited and vatted	oPRP 6.2 Control of the absence of microorganisms in equipment and tanks.
	CCP 6.1 Control of the safety and purity of additives
Alcoholic fermentation, maceration, vat emptying, pressing and malolactic fermentation	oPRP 7.1 Control of the concentration of ethylocarbamate in fermented must.
	oPRP 7.2 Control of hygiene during racking and pressing operations.
	oPRP 7.3 Control of the cleanliness of pressing equipment.
	CCP 7.1 Control of sulfur dioxide in fermented must.
	CCP 7.2 Control of the purity and safety of yeasts.
	CCP 7.3 Temperature control during fermentation.
	CCP 7.4 Control of the pH of red wine during malolactic fermentation.
Racking, clarification, and filtration	oPRP 8.1 Control of the cleaning procedures of tanks and transfer equipment.
	oPRP 8.2 Control of maintenance and cleaning procedures of the facilities.
	oPRP 8.3 Control of hygiene operations during clarification and filtering operations.
	oPRP 8.4 Control of the absence of cleaning and disinfection products in tanks and equipment.
	oPRP 8.5 Control of the absence of foreign elements from the filters in red wine.
	CCP 8.1 Control of the purity and safety of agents used as clarifiers in red wine.
	CCP 8.2 Control of the absence of residues of agents used as clarifiers in red wine.
Cold stabilization	CCP 9.1 Control of limit concentrations of metals (traces of As, Cu, Pb) in red wine.
	CCP 9.2 Control of the additives used are those allowed by current food legislation.
Bottling and labelling	oPRP 10.1 Control of bottle cleaning procedures.
	oPRP 10.2 Control of maintenance and cleaning procedures of the red wine bottling line.
	oPRP 10.3 Control of the correct coding of the labels used on the bottles.
	oPRP 10.4 Control of correct allergen information on labels used on bottles.
	CCP 10.1 Microbiological control of the bottling line of red wine and bottles.
	CCP 10.2 Microbiological control of the cork stopper or similar used for closing the bottles.

Lopez-Santiago (2022) demonstrated that the presence of traces of heavy metals and metalloids in grapes and wines were inadequately controlled CCPs in wineries [27].

These metals and metalloids contamination hazards correspond to CCP 3.1, which involves controlling the presence of metals and metalloids (Cd, Pb, As) in grapes at the Harvest reception stage in the winery, and CCP 9.1, which focuses on controlling the limit concentrations of metals (traces of As, Cu, Pb) in red wine during the Cold stabilization stage in the flow diagram of the young wine-making process as shown in Fig. 1.

According to Lopez-Santiago, fifty percent of the wineries exhibited a complete lack of control over the contamination hazards of arsenic, lead and cadmium in grapes and wines [27].

Herce-Pagliai determined that the concentration of arsenic in Spanish wines ranged from 2.1 to 14.6 μg/L, and the average total arsenic concentration was similar across all wine samples [28]. A review study by the Organisation Internationale de la Vigne et du Vin (OIV) showed that Spanish wines consistently comply with lead concentration limits. The study analyzed sixty-five white and red wines obtaining that lead concentration was below 0.05 mg/L [29].

International and national regulations establish maximum allowable levels of heavy metals and metalloids in grapes and wines to prevent toxicity issues for consumers. The European legislative framework sets maximum permitted concentrations levels for arsenic, lead and cadmium, along with guidelines for monitoring these levels [[30], [31], [32], [33], [34]]**.** According to OIV, the maximum acceptable limits for certain metals in wine are 0.2 mg/L for arsenic, 0.01 mg/L for cadmium, 0.15 mg/L for lead [35]. German legislation sets 0.1 mg/L for arsenic, 0.01 mg/L for cadmium, and 0.3 mg/L for lead, while Italian legislation sets it at 0.3 mg/L [36].

The control of metals and metalloids in grapes and wines is achieved through analytical methods recommended by the OIV, primarily based on atomic absorption spectrometry due to its selectivity, sensitivity, and ability to directly measure these elements. Graphite furnace atomic absorption spectrometry (GFAAS) or electrothermal atomization (ETAAS) are used for arsenic, cadmium, and lead analysis. GFAAS allows detection limits to be lowered to the parts per billion (ppb) range with relative simplicity and eliminating the need for prior extraction techniques [37]. Table 2 presents the OIV recommended methods for determining Arsenic [38], Cadmium [39] and Lead [40] in wines and must.

**Table 2:** International methods to determine arsenic, cadmium, and lead in wines and must recommended by the OIV.

Element	Method	Summary
Arsenic	OIV-MA-AS323-01A Determination of arsenic in wine by atomic absorption spectrometer	This method is a recommended procedure for the determination of arsenic in wine using GFAAS and hydride generation. The equipment required includes glassware, a water bath, filters, and a spectrophotometer with specific instrumental parameters. Various reagents such as nitric acid, potassium iodide, hydrochloric acid, sodium borohydride, and sodium hydroxide are used. Calibration standards are prepared, and the sample preparation involves evaporation, addition of potassium iodide and hydrochloric acid, and filtration. The determination is performed by introducing the borohydride solution, hydrochloric acid solution, and sample solution into the peristaltic pump. Calibration standards and samples are analyzed, and the software establishes the arsenic concentration. Quality control is maintained by including control samples from the Bureau Communautaire de Référence.
Cadmium	OIV-MA-AS322-10 Determination of arsenic in wine by atomic absorption spectrometer	This method is a recommended procedure for the direct determination of cadmium in wine using atomic absorption spectrometer. The apparatus required includes an atomic absorption spectrophotometer with a graphite furnace, background correction, and a recorder, as well as specific glassware and a cadmium hollow cathode lamp. Various reagents, including phosphoric acid, ethylenediamine tetra-acetic acid solution, buffer solution, and Eriochrome black T, are used in the analysis. The concentration of cadmium sulfate is verified through titration with ethylenediamine tetra-acetic acid solution. The procedure involves sample preparation, preparation of calibration standards, and determination using specific furnace programming and atomic absorption measurements. The results are expressed as the concentration of cadmium in micrograms per liter of wine.
Lead	OIV-MA-F1-10 Specific methods Type IV method Annex D: Heavy metals D.1 Determination of lead level by Electrothermal Atomic Absorption Spectrophotometry	This is an ETAAS technique for determining lead levels in grape sugar samples. It provides instructions on reagents, equipment, calibration, safety precautions, and sampling. The method covers a lead concentration range of 10 μg/kg to 200 μg/kg. The use of lead-free reagents is emphasized, and calibration curves are used to calculate lead concentrations. The apparatus required includes an atomic absorption spectrometer with specific settings. The specific settings for the ETAAS method include using an electrothermal atomizer, a hollow-cathode lamp for lead, a background noise correction device, and adjusting temperature and gas flow rates for optimal measurement range and sensitivity. Zeeman background noise correction is preferred to reduce interference. Precision parameters for repeatability and reproducibility are provided for lead concentrations below 150 μg/kg.

Within this framework, the primary aim of this research is to assess the effectiveness of wineries in managing Critical Control Points (CCPs) associated with contamination risks posed by arsenic, cadmium, and lead in grapes and wines. Additionally, the study aims to develop a methodology for evaluating their advancement, incorporating the use of performance indicators within the HACCP plan to highlight the element of training as a corrective action. Furthermore, the study aims to identify the challenges that impede achieving adequate control.

## 2 Materials and methods

### 2.1 Study design

The research study design was proposed by conducting a survey and its later analysis using right statistical methods. The sample was selected among Spanish wineries from different wine regions. During last half of 2022, the survey was conducted, and then, SPSS Windows software SPSS was used to analyse the data (IBM Corp. Released 2020. IBM SPSS Statistics for Windows, Version 27.0. Armonk, NY, USA: IBM Corp.). The calculated statistics were frequencies and central position values. Non-parametric tests were estimated obtaining Spearman correlation coefficient (ρ) and Kendall's Tau coefficient (τ) for nonparametric data, with a significance level of p < 0.01. Non-parametric Mann-Whitney *U* Test for two independent samples were applied, with a significance level of p < 0.05.

### 2.2 Sample selection

Spain had approximately 4133 wineries in 2020 [41] and one hundred and one Wine Protected Designation of Origin (WPDO) [42]. One hundred-thirty-nine wineries were selected from different WPDOs for this research. The sampling methodology selected was the non-probabilistic method [43,44]. Researchers used previous information to make the sample selection, instead of random selection [45]. The criteria for configuring the sample were that there was diversity in wineries′ sizes according to their annual wine production, wineries must belong to a WPDO, and has been HACCP implemented. Wineries were asked about their performance in controlling the risk of arsenic, lead, and cadmium contamination critical control point in the winemaking.

The questionnaire was sent twice to all the wineries in the sample, and additionally, the questionnaire was sent once again to fifty of them through the 'Contact' section of their website. Thirty-two wineries answered the questionnaire, which represents 23 % of the wineries sampled.

### 2.3 Survey preparation

**Fig. 2:**
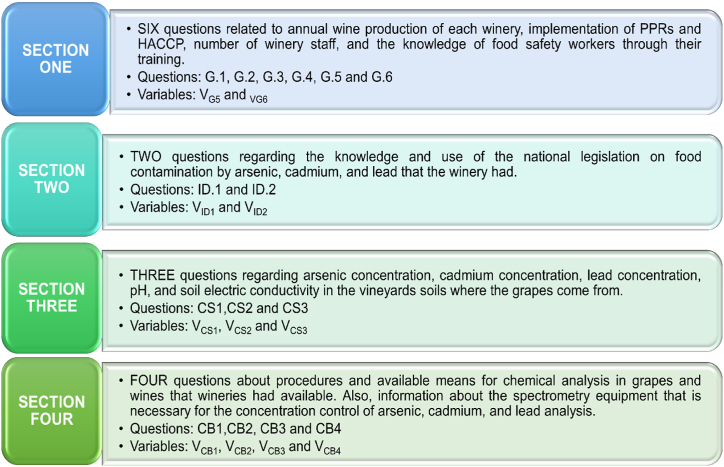
Structure of the questionnaire that encompassing four sections with its specific content.

The survey design consisted of a questionnaire divided into four sections, with a total of fifteen closed-ended questions. The questions were developed based on previous research studies [2,3,10,19,29,46,47]. The questionnaire can be found in Appendix A. This type of questionnaire is commonly used in causal, descriptive, and conclusive research [48]. Fig. 2 illustrates the questionnaire structure, including the contents of each section, and the questions and variables in each section.

Question G.1 determined winery size based on yearly wine production and assigned winery groups to cover all responses. Questions G.2 and G.3 are dichotomous (yes/no) questions about PPR and HACCP implementation and, Question G.4 inquired about the number of winery staff.

Question G.5 focused on the number of workers trained in Good Manufacturing Practices (GMP). It utilized a qualitative scale and a quantitative variable represented by a Likert scale (ranging from 0.33 to 1). Variable V_G5_ indicated the level of GMP training among workers, with values of 0.33 representing "No workers have GMP training," 0.66 representing "More than half of workers have GMP training," and 1 representing "All workers have GMP training."

Similarly, question G.6 asked about the number of workers trained in the control and monitoring of CCPs. It also utilized a qualitative scale and a quantitative variable represented by a Likert scale (ranging from 0.33 to 1). Variable V_G6_ indicated the level of workers trained in CCPs, with values of 0.33 representing "No workers have training in control and monitoring of CCPs," 0.66 representing "More than half of workers have training in control and monitoring of CCPs," and 1 representing "All workers have training in control and monitoring of CCPs."

Question ID.1 was a multiple-choice question assessing winery performance in identifying legislation related to arsenic, cadmium, and lead. It was coded using a Likert scale, with the variable V_ID1_ (ranging from 0.33 to 1) representing winery performance regarding legislation identification. Question ID.2 was a dichotomous (yes/no) question asking about winery identification of updated information from the Spanish Agency for Food Safety and Nutrition (AESAN) on heavy metals and metalloids food risk. Variable V_ID2_ took values of 0 for "No" and 1 for "Yes."

Question CS1 was a dichotomous (yes/no) question about winery information regarding vineyard soil physical and chemical analysis. Variable V_CS1_ took values of 0 for "No" and 1 for "Yes." Question CS2 was a dichotomous (yes/no) question about winery information regarding fertilizer use in vineyard soils. Variable V_CS2_ took values of 0 for "No" and 1 for "Yes." Question CS3 was a multiple-choice question with eight options regarding winery information on soil chemical properties. Variable V_CS3_ calculated the cumulative value (0.125) assigned to each selected option, indicating the level of winery knowledge regarding specific soil chemical properties.

Question V_CB1_ was a dichotomous (yes/no) question about whether the winery had its own laboratory for chemical analyses. Variable V_CB1_ took values of 0 for "No" and 1 for "Yes." Question C_B2_ was a dichotomous (yes/no) question about whether the winery used an external laboratory for chemical analyses. Variable V_CB2_ took values of 0 for "No" and 1 for "Yes." Question CB3 was a dichotomous (yes/no) question about whether the winery had its own atomic absorption spectrometer and staff for metal analyses. Variable V_CB3_ took values of 0 for "No" and 1 for "Yes”. Question CB4 was a dichotomous (yes/no) question about whether the winery used an external laboratory for metal trace analyses. Variable V_CB4_ took values of 0 for "No" and 1 for "Yes”. Finally, there was a multiple-choice question about the job position of the survey respondent.

We have formulated three hypotheses to be examined in this study. Hypothesis one (H1) proposed that workers who received adequate GMP and CCP training demonstrate a satisfactory performance level of CCP controlling in the wineries. Hypothesis two (H2) stated that legislation performance component regarding to its identification and updating about the contamination risk posed by arsenic, cadmium, and lead has reached a mature level in the wineries. Hypothesis three (H3) proposed that wineries possess information about the concentrations of arsenic, cadmium, and lead in the vineyard soils from which grapes (raw material) are harvested and that wineries have adequate spectrometric equipment for their identification.

## 3 Results

**Fig. 3:**
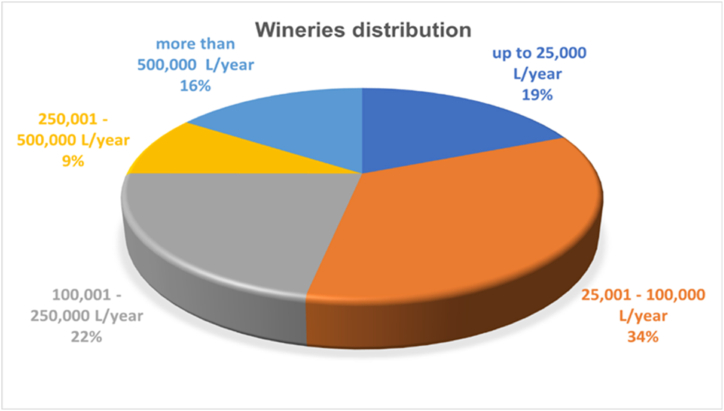
Wineries sample distribution according to their yearly wine production (L/year).

Fig. 3 shows the wineries distribution in five groups according to their answers about their yearly wine production.

Regarding the FSMS implementation, 96.9 % of the wineries had implemented PRPs according to food hygiene legislation, and a 93.8 % of the wineries had implemented a HACCP.

### 3.1 Performance of wineries in relation to food safety training component

Table 3 shows the results for the percentage of workers trained in good manufacturing practices in winemaking (GMP), the percentage of workers trained in the monitoring and CCPs, and number of workers median and arithmetic mean by type of winery. All workers in wineries with annual production over 250,001 L/year have received GMP training and all workers in wineries with annual production over 500,000 L/year have received CCPs training. Although some wineries producing between 25,001–250,000 L/year have all workers without GMP or CCPs training, it can be said that most of the wineries have some GMP-trained and CCPs-trained workers.

**Table 3:** GMP workers training and CCPs workers training by type of winery.

Wine Annual Production L/year	Percentage of Wineries Over Total	GMP Workers training (%)			CCPs Workers training (%)			Number of workers	
		All	More Than 50 %	None	All	More than 50 %	None	Median	Arithmetic mean
up to 25,000	18.8	66.7	33.3	0.0	66.7	33.3	0.0	2.5	2.3
25,001–100,000	34.4	60.0	20.0	20.0	40.0	50.0	10,0	4.0	3.3
100,001–250,000	21.9	28.6	57.1	14.3	28.6	57.1	14.3	5.0	5.1
250,001–500,000	9.4	100.0	0.0	0.0	66.7	33.3	0.0	7.0	7.3
more than 500,000	15.6	100.0	0.0	0.0	100.0	0.0	0.0	10.0	21.7
Total wineries Percentage	100.0	65.6	21.9	12.5	53.1	37.5	9.4	4.0	6.1

Spearman correlation coefficient (ρ) is 0.686 and Kendall's Tau coefficient (τ) is 0.653 between variables V_G5_ and V_G6_. It shows there is a positive correlation between the GMP Workers Training and CCPs Workers Training.

Results show that as the winery gets bigger according to its yearly wine production, it has more workers trained in GMP and CCPs. However, the percentage of trained workers is also high in smaller wineries. This is due to the number of workers ranging between two and three in this winery group, and therefore having trained a worker already reaches values of fifty percent**.** This finding is in agreement with the study conducted by Lee J.C. et al. [49], which identified significant increases in the application of GMP, GHP, and equipment design prerequisites, as well as all HACCP systems, in European companies**.**

In general, wineries train a higher percentage of workers in GMP than in CCPs. One in two wineries has half of its workers untrained in CCPs. This is a difficulty for identification and control of CCP related to the risk of contamination by arsenic, cadmium, and lead during the winemaking process. Wineries that supply training to their workers do so in both GMP and CCPs.

A quantitative analysis of food safety worker training (FSWT) was performed based on an indicator defined by equation (1) [50,51]:

(1) $$W_{fswt}=\left(V_{G5}+V_{G6}\right)/n$$

Where.

• $W_{fswt}$ is aggregated FSWT variable for each winery,
• $V_{G5}$ is variable that stands for the level of workers trained in GMP, and takes values 0.33, 0.66 or 1.
• $V_{G6}$ is variable that represent level of workers trained in CCP, and takes values 0.33, 0.66 or 1.
• n is number of variables that has been aggregated, and its value is 2.

Obtaining FSWT Indicator ($I_{fswt}$) for each group of wineries according to their yearly wine production size by equation (2):

(2) $$I_{fswt}=\frac{\sum_{i=1}^{m}W_{fswt}}{m}$$

Where.

• $I_{fswt}$ is FSWT Indicator for each group of wineries according to yearly wine production,
• $W_{fswt}$ is FSWT variable for each winery,
• m is number of wineries of related group.

This indicator is dimensionless and expresses the grade of progress achieved regarding food safety worker training in each of the winery groups, according to their annual production. The grades of progress are defined as Star ($I_{fswt}$ between 0 and 0.33), In progress ($I_{fswt}$ between 0.34 and 0.67), and Maturity ($I_{fswt}$ between 0.67 and 1). $I_{\text{fswt}}$ values for winery size group regarding to their annual wine production are showed in Table 6.

### 3.2 Performance of wineries in relation to heavy metal and metalloids food contamination risk legislation component

Second section results are collected in Table 4. This table shows the heavy metal and metalloids food contamination risk (HMFCR) legislation identification and HMFCR legislation updating the through National Agency (AESAN) by type of winery.

**Table 4:** Heavy Metal Food Contamination Risk (HMFCR) legislation identification and HMFCR legislation updating through National Agency (AESAN) by type of winery.

Wine Annual Production L/year	Percentage of Wineries Over Total	HMFCR legislation identification (%)				HMFCR Legislation updating through AESAN (%)	
		As	Cd	Pb	None	Yes	No
up to 25,000	18.8	33.3	33.3	50.0	50.0	50.0	50.0
25,001–100,000	34.4	33.3	33.3	33.3	66.7	33.3	66.7
100,001–250,000	21.9	28.6	28.6	28.6	71.4	28.6	71.4
250,001–500,000	9.4	66.7	66.7	66.7	33.3	66.7	33.3
more than 500,000	15.6	66.7	66.7	66.7	33.3	66.7	33.3
**Total wineries Percentage**	**100.0**	**31.2**	**31.2**	**37.5**	**62.5**	**37.5**	**62.5**

There is a low wineries percentage that have identified arsenic, cadmium, and lead contamination risk legislation. Rates are higher in wineries with wine production over 250,000 L/year. In this case, two out of three wineries have identified arsenic, cadmium, and lead legislation. Wineries between 100,001–250,001 have the lowest rate (28.6 %).

National Agency thar integrates and performs the functions related to food safety within the competence framework of the General Administration of Spain is the Agencia Española de Seguridad Alimentaria y Nutrición (AESAN) [52]. Information on applicable arsenic, cadmium and lead contamination food risks legislation is available and updated on the AESAN website [53]. AESAN information is used by only one-third of small to medium-sized wineries (up to 250,000 L/year). information. Bigger wineries (over 250,001 L/year) have a better rate (66.7 %), but it is still insufficient. HMFCR legislation is not clearly identified in wineries, and this occurs especially in wineries with annual wine production under 250,001 L/year. The Spearman correlation coefficient (ρ) is 0.894 and Kendall's Tau coefficient (τ) is 0.873 between variables V_ID1_ and V_ID2_. It shows there is a strong positive relationship between the identification of HMFCR legislation and it is and updating through AESAN. Besides, correlation coefficients have been calculated for V_ID1_ and V_ID2_. The Spearman correlation coefficient (ρ) is 0.413 and Kendall's Tau coefficient (τ) is 0.393. A positive correlation exists between the workers′ CCPs training and the HMFCR legislation identification in the wineries.

A quantitative analysis of legislation identification and updating (LIU) was performed based on an indicator defined by equation (3):

(3) $$W_{\text{liu}}=\left(V_{ID1}+V_{ID2}\right)/n$$

Where.

• $W_{liu}$ is aggregated LIU variable for each winery,
• $V_{\text{ID}1}$ is variable that stands for winery performance about legislation identification about arsenic, cadmium, and lead, and takes values 0.33, 0.66 or 1.
• $V_{\text{ID}2}$ is variable that represented winery performance about update legislation information through AESAN and takes values 0 or 1.
• n is number of variables that has been aggregated, and its value is 2.

Obtaining LIU Indicator ($I_{liu}$) for each group of wineries according to their yearly wine production size by equation (4):

(4) $$I_{l\text{iu}}=\frac{\sum_{i=1}^{m}W_{liu}}{m}$$

Where.

• $I_{liu}$ is LIU Indicator for each group of wineries according to yearly wine production,
• $W_{liu}$ is LIU variable for each winery,
• m is number of wineries of related group.

This indicator is dimensionless and expresses the grade of progress achieved regarding the analysis of legislation identification and updating in each of the winery groups, according to their annual production. The grades of progress are defined as Star ($I_{liu}$ between 0 and 0.33), In progress ($I_{liu}$ between 0.34 and 0.67), and Maturity ($I_{liu}$ between 0.67 and 1). $I_{\text{liu}}$ values for winery size group regarding to their yearly annual wine production are showed in Table 6.

### 3.3 Performance of wineries in relation to chemical analysis of the vineyards regarding to arsenic, cadmium, and lead

Most wineries have data about vineyard soils′ physical and chemical analysis, and fertilizer information used in the vineyard soils. Spearman correlation coefficient (ρ) between $V_{\text{CS}1}$ and $V_{\text{CS}2}$ is 0.686. This positive correlation demonstrates that wineries that possess information about the physical and chemical analysis of vineyard soil tend to also have information about the fertilizers used in those vineyard soils.

**Fig. 4:**
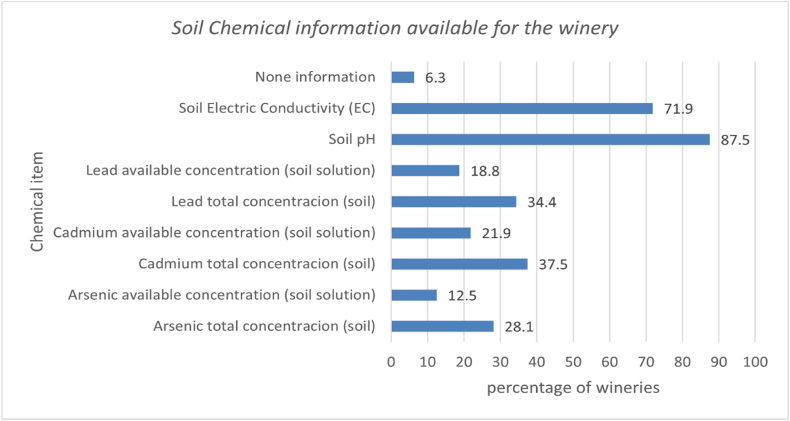
Percentage of wineries that have information related to the soil chemical analysis of the vineyards regarding to arsenic, cadmium and lead concentrations, Soil pH, and EC.

The percentage of wineries that have information related to the soil chemical analysis of the vineyards where the grapes come from shown in Fig. 4.

However, this information is mostly about soil pH (87.5 %) and soil electric conductivity (71.9 %). The number of wineries that have chemical information about arsenic, cadmium, and lead concentrations decreases considerably. Table 5 shows data on the percentage of wineries, segmented according to their level of annual wine production, which have information regarding arsenic, cadmium, and lead total/available concentrations.

**Table 5:** of wineries that have information related to the soil chemical analysis of the vineyards regarding to arsenic, cadmium, and lead concentrations, by type of winery.

Wine Annual Production L/year	Percentage of Wineries Over Total	Total concentration (soil) (%)			Available concentration (Soil solution) (%)			None
		As	Cd	Pb	As	Cd	Pb	
up to 25,000	18.8	33.3	16.6	16.6	0.0	0.0	0.0	0.0
25,001–100,000	34.4	18.2	27.3	27.3	18.2	27.3	27.3	9.1
100,001–250,000	21.9	14.3	42.9	28.6	0.0	28.6	14.3	0.0
250,001–500,000	9.4	33.3	66.7	66.7	33.3	66.7	66.7	0.0
more than 500,000	15.6	40.0	40.0	40.0	0.0	0.0	0.0	40.0
**Total wineries Percentage**	**100.0**	**28.1**	**37.5**	**34.4**	**12.5**	**21.9**	**18.8**	**6.3**

**Table 6:** Wineries performance indicators and grade of progress, by type of winery.

Wineries size	$I_{fswt}$	Grade of progress	$I_{liu}$	Grade of progress	$I_{ccp-Mchem}$	Grade of progress
**Up to 25,000 L/year**	0.89	Maturity	0.44	In progress	0.05	Start
**25,001–100,000 L/year**	0.77	Maturity	0,15	Start	0.08	Start
**100,001–250,000 L/year**	0.78	Maturity	0.28	Start	0.51	In progress
**250,001–1,000,000 L/year**	0.94	Maturity	0.66	In progress	0.56	In progress
**More than 500,000 L/year**	0.93	Maturity	0.60	In progress	0.42	In progress

One out of every three wineries possess data regarding the cumulative levels of arsenic, cadmium, and lead concentrations in the soil. Besides, wineries percentage decreases when the information is about soil solution concentrations of arsenic, cadmium, and lead. Hence, one in ten possesses data concerning the presence of arsenic in vineyard soils, while two in ten wineries have information pertaining to the concentrations of cadmium and lead in the same soil samples.

Information data about total cadmium and lead concentrations are the ones that most have the wineries, especially the largest wineries (over 250,001 L/year). Instead of the information about total arsenic concentration, it says that it is an extremely low percentage in all winery groups. The percentages of wineries relating to the information about the concentrations of arsenic, cadmium, and lead in the soil solution are lower those relating to the total concentration. It is the information related to the concentration of cadmium available in the soil solution that presents the highest percentage, being 66.7 in the wineries between 250,001–500,000 L/year and 28.6 in the wineries between 100,001–250,000 L/year. The lack of information on arsenic, cadmium, and lead soil concentrations is a difficulty for the CCP controlling as it impedes an adequate assessment of the risk that grapes used for winemaking may have been contaminated during cultivation or harvest.

A high percentage of wineries (78.1 %) have their own laboratory in their facilities to make chemical analyses of grapes and wines. Two out of every ten wineries do not have their own laboratory. In this case, most of them (92.3 %) use an external laboratory service to make chemical analyses of grapes and wines. In this context, control of arsenic, cadmium, and lead in grapes and wines is made by analytical techniques based on atomic absorption spectrometry. Only one out of every ten wineries possess the necessary technological equipment and qualified staff capable of conducting heavy metal analysis using atomic absorption spectrometry. A high percentage of wineries (93.8 %) do not have them. Thirty-five-point-two percent of wineries without atomic absorption spectrometry equipment and qualified staff use external laboratories to analyse about heavy metals and metalloids concentration in grapes and wines. This result shows that the lack of technical means for qualitative and quantitative analysis in wineries is a barrier to good performance related to controlling arsenic, cadmium and lead contamination risk in grapes and win es.

A quantitative analysis of arsenic, cadmium, and lead critical control point chemical analysis performance (CCP-MCHEM) was evaluated based on an indicator defined by equation (5):

(5) $$W_{\text{ccp}-\text{Mchem}}=\left(V_{CS3}+V_{CBrx}+V_{CBry}\right)/n$$

Where.

• $W_{\text{ccp}-\text{Mchem}}$ is the aggregated CCP-MCHEM variable for each winery,
• $V_{\text{CS}3}$ is variable that stood for chemical information about arsenic, cadmium, and lead concentrations in soil that winery had. $V_{CS3}=\sum_{j=1}^{8}a_{j},$ $a_{j}$ is each item of this multiple-choice question (yes = 0.125, no = 0),
• $V_{\text{CBrx}}$ is variable that represented winery capacity to hold chemical analysis by their own or external means. $V_{\text{CBrx}}=V_{\text{CB}2}$ if $V_{\text{CB}1}=0$, otherwise $V_{\text{CBrx}}=V_{\text{CB}1}$.
• $V_{\text{CBry}}$ is variable that stood for winery capacity to hold arsenic, cadmium and lead chemical analysis by their own or external means. $V_{\text{CBry}}=V_{\text{CB}4}$ if $V_{\text{CB}3}=0$, otherwise $V_{\text{CBry}}=V_{\text{CB}3}$.
• n is the number of variables that has been aggregated, and its value is 3.

Obtaining CCP-MCHEM Indicator ($I_{\text{ccp}-\text{Mchem}}$) for each group of wineries according to their yearly wine production size by equation (6):

(6) $$I_{\text{ccp}-\text{Mchem}}=\frac{\sum_{i=1}^{m}W_{\text{ccp}-\text{Mchem}}}{m}$$

Where.

• $I_{\text{ccp}-\text{Mchem}}$ is CCP-MCHEM Indicator for each group of wineries according to yearly wine production,
• $W_{\text{ccp}-\text{Mchem}}$ is CCP-MCHEM variable for each winery,
• m is number of wineries of related group.

This indicator is dimensionless and expresses the grade of progress achieved regarding to arsenic, cadmium, and lead critical control point chemical analysis performance in each of the winery groups, according to their annual production. The grades of progress are defined as Star ($I_{\text{ccp}-\text{Mchem}}$ between 0 and 0.33), In progress ($I_{\text{ccp}-\text{Mchem}}$ between 0.34 and 0.67), and Maturity ($I_{\text{ccp}-\text{Mchem}}$ between 0.67 and 1). $I_{\text{ccp}-\text{Mchem}}$ values for winery size group regarding to their annual wine production are showed in Table 7.

**Table 7:** Relationship questions among wineries performance variables, their imply variables and MWU results.

Question	Variables	Non-parametric Mann-Whitney *U* Test	Result
Did grade of progress of training component differ according to whether wineries conducted identification and updating arsenic, cadmium, and lead contamination risk legislation available in AESAN or did not?	$W_{\text{fswt}}$ as a dependent variable V_ID2_ as an independent variable which stood for two groups (V_ID2_ = 0, V_ID2_ = 1).	Z = −2.673 Bilateral significance = 0.008 Exact significance = 0.013 Monte Carlo significance = 0.009, lower limit = 0.007, upper limit = 0.011 p < 0.05	Rejected hypothesis null. (Differed)
Did grade of progress of training component differ according to wineries capacity to do arsenic, cadmium and lead chemical analysis by their own or external means or did not?	$W_{\text{fswt}}$ as a dependent variable $V_{\text{CBry}}$ as an independent variable which stood for two groups (V_CBry_ = 0, V_CBry_ = 1).	Z = −0.555 Bilateral significance = 0.579 Exact significance = 0.616 Monte Carlo significance = 0.598, lower limit = 0.598, upper limit = 0.607 p < 0.05	Accepted hypothesis null. (Did not differ)
Did grade of progress of the legislation component differ according to wineries capacity to do arsenic, cadmium and lead chemical analysis by their own or external means or did not?	$W_{liu}$ as a dependent variable $V_{\text{CBry}}$ as an independent variable which stood for two groups (V_CBry_ = 0, V_CBry_ = 1).	Z = −2.506 Bilateral signature = 0.012 Exact signature = 0.029 Monte Carlo significance = 0.013, lower limit = 0.011, upper limit = 0.016 p < 0.05	Rejected hypothesis null. (Differed)
Did grade of progress of the legislation component differ according to whether wineries conducted identification and updating arsenic, cadmium, and lead contamination risk legislation available in AESAN or did not?	$W_{\text{liu}}$ as a dependent variable V_ID2_ as an independent variable which stood for two groups (V_ID2_ = 0, V_ID2_ = 1).	Z = −5.355 Bilateral signature = 0.001 Exact signature = 0.001 Monte Carlo significance = 0.000, lower limit = 0.000, upper limit = 0.000 p < 0.05	Rejected hypothesis null. (Differed)
Did grade of progress of the analysis component differ according if winery had information related to vineyards soil physical and chemical analysis or did not?	$W_{\text{ccp}-\text{Mchem}}$ as a dependent variable $V_{\text{CS}1}$ as an independent variable which stood for two groups ($V_{\text{CS}1}$ = 0, $V_{\text{CS}1}$ = 1).	Z = −3.288 Bilateral signature = 0.001 Exact signature = 0.001 Monte Carlo significance = 0.000, lower limit = 0.000, upper limit = 0.000 p < 0.05	Rejected hypothesis null. (Differed)
Did grade of progress of the analysis component differ according if winery had fertilizer information used in vineyard soils or did not?	$W_{\text{ccp}-\text{Mchem}}$ as a dependent variable $V_{\text{CS}2}$ as an independent variable which stood for two groups ($V_{\text{CS}2}$ = 0, $V_{\text{CS}2}$ = 1).	Z = −3.288 Bilateral signature = 0.001 Exact signature = 0.001 Monte Carlo significance = 0.000, lower limit = 0.000, upper limit = 0.000 p < 0.05	Rejected hypothesis null. (Differed)

### 3.4 Matrix and graph of the grade of progress in wineries generated by the performance indicators

The three performance indicators allow to determine the degree of progress that each group of wineries has reached related to the control that they conduct on the contamination risk of arsenic, cadmium, and lead in grapes and wines. Table 6 shows values I_fswt_, I_liu_ and I_ccp-Mchem_ by winery sizes groups and grade of progress.

Effectiveness in conducting risk control is divided into three components: the training component, the legislation component, and the analysis component.

**Fig. 5:**
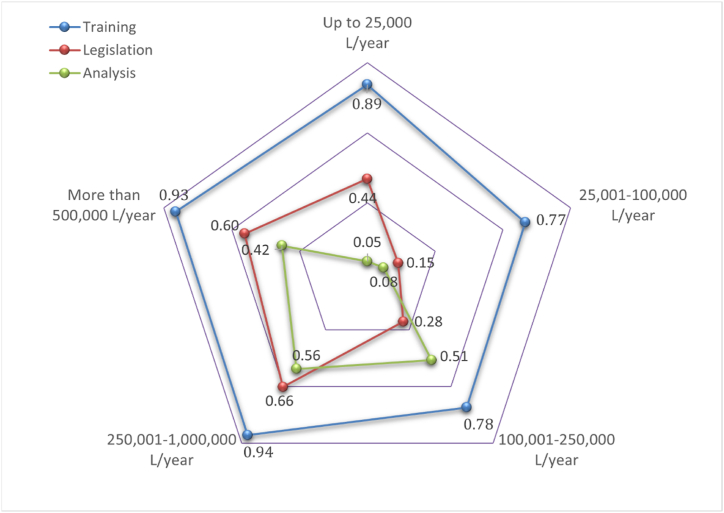
Performance level in each component by annual winery production.

Each indicator stands for the degree of progress on a component. $I_{fswt}$ is performance indicator that shows progress in the training component. $I_{liu}$ is the indicator that shows progress in the legislation component and $I_{\mathrm{ccp}-\mathrm{Mchem}}$ is the indicator that shows progress in analysis component. Components performance level by each winery sizes group on the contamination risk by arsenic, cadmium and lead in grapes and wines in Fig. 5.

Training is the component with the greatest maturity while analysis is the component with the least maturity regarding to this contamination risk control. Wineries below 250,000 L/year are in the starting performance level of the analysis component, and wineries between 25,001 and 250,000 L/year are in the starting performance level of the legislation component.

Non-parametric Mann-Whitney U Tests (MWU) were applied to find relationships among wineries performance variables. Table 7 shows relationship questions, their imply variables and MWU results. Grade of progress of training component differed according to whether wineries conducted identification and updating the legislation related to risk of arsenic, cadmium, and lead contamination available in AESAN, but it was not different regarding to whether wineries had capacity to do arsenic, cadmium and lead chemical analysis by any means.

Grade of progress of the legislation component was different according whether wineries had capacity to do arsenic, cadmium and lead chemical analysis by any means and differed regarding to if wineries conducted identification and updating arsenic, cadmium, and lead contamination risk legislation available in AESAN or did not.

Grade of progress of the analysis component differed according if winery had information regarding to vineyards soil physical-chemical analysis and, according to if winery had fertilizer information used in vineyard soils or had not.

## 4 Discussion

An evaluation of how wineries are managing critical control point about controlling the contamination risk by arsenic, cadmium and lead in their winemaking processes is essential to prevent poisoning and diseases in consumers. Studies have identified health problems caused by As, Cd, and Pb [54,55].

Fertilizers or the environment are sources of arsenic, cadmium and lead that can contaminate grapes and wines used in wine production [9,55,56].

### 4.1 GMP and CCP workers training

FSMS are tools to accomplish with food hazards control as heavy metals and metalloids traces in grapes and wines. There is an important level of implementation of FSMS in wineries. 96.9 % of wineries have a Prerequisites Programmes and 93.8 % have HACCP. Besides, workers trained in GMP and CCPs have a satisfactory level for all wineries.

The study reveals that hypothesis 1 (H1) is true. The training component has reached an adequate maturity level of performance. 65.6 % of wineries have all their workers trained in GMP and 53.1 % of wineries have all their workers trained in CCPs. These percentages arise to 100 % in wineries with annual production over 500,001 L/year. Results show that wineries′ workers who receive training in GMP, also receive training in CCPs. Similarly, other related studies focusing on controlling OTA mycotoxin contamination in wines have also demonstrated that training in GMP and CCPs has been identified as a significant contributing factor to the successful prevention of such contamination [24]. Our findings are consistent with the research conducted by Lee, J.C. et al. [49], wherein the importance of aspects related to food safety culture is underscored, particularly about human factors and specialized training.

### 4.2 Knowledge and application of food safety legislation

Hypothesis 2 (H2) is not fulfilled. Legislation component is still in progress level regarding performance maturity in four up five wineries groups. European legislation on food safety related to contamination control risk from arsenic, cadmium and lead is accessible. Study shows that only one out three wineries with annual production less than 250,000 L/year has identified and updated the HMCR legislation. The HCMR legislation identification percentage is 66.7 % in wineries with annual production over 250,001 L/year. Two out of three wineries that have the HCMR legislation identified conduct its update through the information available in the Food Safety Spanish Agency (AESAN). Mere publication of food risk information on the AESAN website is not enough for wineries to incorporate it into their control system. Two out of three wineries do not have updated legislation and do not use the information provided by AESAN.

Most of the wineries that have identified the HCMR legislation, also have their staff trained in CCPs. Identification of food risk control legislation is different according to the wine production level of the wineries and their workers′ knowledge. The public administration does not provide sufficient references and means for wineries to properly develop and implement the control of CCP related to the risk contamination of arsenic, cadmium, and lead. Our result is aligned with Vela A.R. & Fernández [56], that demonstrated public administrations received a low score from companies regarding to the references provided by the administration (reports, books, and articles) to facilitate the development and implementation of FSMS. Matches with the alcoholic beverage industry's weakness in compliance with food safety legislation demonstrated by Kourtis L.K [57].

Charlotte Yap [58] found that knowledge about the general principles of food safety and its requirements is low in small and medium-sized agrifood businesses. This often leads to regulatory requirements being underestimated and not considered. This lack of knowledge regarding the risk of heavy metal and metalloid contamination in food is a weakness observed in wineries' control over their winemaking processes.

Our findings also align with the study conducted by Allam, M. et al. [59], which identified areas where organic food producers and processors in several European countries require further guidance and support in food safety, particularly in their proficiency in performing hazard analysis and creating documents and records following HACCP principles.

### 4.3 Analysis and availability of chemical data related to the control of contamination by As, Cd, and Pb

Hypothesis 3 (H3) is not accomplished. Analysis is the lower performance component for all winery groups, except 100,000 to 250,000 L/year. We found that many wineries have not information about arsenic, cadmium, and lead concentrations in the vineyards soils, and the wineries percentage that owns this information is different according to their wine annual production level. Nine of ten wineries have information about soil physical and chemical characteristics, such as pH and EC, and information about the fertilizers used in the vineyards. However, these values strongly decrease about the arsenic, cadmium, and lead concentrations in these soils. The proportion of wineries equipped with data on the cumulative concentrations of arsenic, cadmium, and lead is remarkably low. However, these percentages become even more diminished when considering the availability of information concerning specific arsenic, cadmium, and lead available concentrations in the soil. Out of the ten wineries, two wineries have this chemical concentration information about cadmium and lead, and one winery has this chemical concentration information about arsenic. A deficient control of these the arsenic, cadmium, and lead in soils implies that food hazards as the appearance of heavy metal traces in grapes or wine may occur [60,61]. The wineries use techniques based on atomic absorption spectrometry for the identification of the arsenic, cadmium, and lead in the grapes and wines. This technique is recommended by the OIV. However, there is a lack of this spectrometry equipment in the wineries own laboratories, so they need to use external analytical services. Results show that half of the wineries (48.59 %) use atomic absorption spectrometry analysis to detect the presence of arsenic, cadmium, and lead in the grapes and wines through external analytical services. Courtney K. Tanabe (2019) identified the absence of on-site chemical analytical tools as a factor that influences the arsenic content in grapes and wines [62].

### 4.4 Limitation and strength of the study

One of the principal weaknesses of this study lies in the low response rate obtained from the surveyed wineries, potentially impacting the representativeness and generalizability of the data to the entire wine industry. However, a fundamental strength of this research is rooted in the meticulous development of performance indicators based on the questionnaire methodology. This tool provides a robust foundation for data analysis and enables the evaluation of wineries' effectiveness in managing Critical Control Points (CCPs) associated with contamination risks posed by arsenic, cadmium, and lead in grapes and wines.

## 5 Conclusions

This research shows that although most wineries have FSMS implemented, the CCP identification and control related to arsenic, cadmium and lead contamination risk needs to be improved.

Wineries must adequately control the risk of arsenic, cadmium, and lead contamination in the wine production process. To do it, first, wineries must be aware of the need to know, update and implement European legislation. European legislation sets the guidelines to prevent health risks that may arise from the intake of arsenic, cadmium, or lead by setting limits on the admissible concentration of these metals in wines. In addition, wineries′ workers′ training in GMP and CCPs control is a success factor in preventing contamination by arsenic, cadmium, and lead in wines.

A strength in the performance of CCP control aimed at assessing the risk of arsenic, cadmium and lead contamination is high training level of workers about this topic. However, the wineries performance about applicable legislation identification of the heavy metal contamination risk is very low. This constitutes a difficulty for good performance of CCP controlling relative to the risk of contamination by arsenic, cadmium, and lead. In addition, another difficulty for this performance is the lack of information regarding the sources of contamination by arsenic, cadmium, and lead in grapes. Soil analysis data about pH and EC are available to wineries, but data about the concentrations of As, Cd and Pb in the soil are often missing.

Another barrier to a good performance of the CCP related to contamination of grapes by arsenic, cadmium, and lead is the lack of spectrometry equipment in the laboratories of the wineries. Even if external services are hired for spectrometric analysis, the percentage of wineries that perform it is very low. Providing wineries with laboratory spectrometry equipment and human resources to carry out complete chemical analyses of soils, grapes and wines would allow the adequate control of CCP related to contamination risk control by heavy metals and metalloids of their grapes and wines.

## Ethic statement

The authors confirm that the study and data collection comply with all ethics regulations and confirm that informed consent was obtained from the participants in collecting data.

## Funding

This research did not receive any specific grant from funding agencies in the public, commercial or not-for-profit sectors.

## Data availability statement

Data associated with the study has not been deposited into a publicly available repository and data will be made available on request.

## CRediT authorship contribution statement

**Jesús López-Santiago:** Writing - review & editing, Writing - original draft, Validation, Methodology, Formal analysis, Data curation, Conceptualization. **Ana Isabel García García:** Supervision. **Alejandro Gómez Villarino:** Validation, Methodology. **Amelia Md Som:** Writing - review & editing, Validation, Supervision, Methodology. **María Teresa Gómez-Villarino:** Writing - review & editing, Validation, Supervision, Methodology.

## Declaration of competing interest

The authors declare that they have no known competing financial interests or personal relationships that could have appeared to influence the work reported in this paper.

## Appendix A

Analysis of wineries performance about Critical Control Point related to risk control contamination by arsenic, cadmium, and lead in grapes/wines during winemaking.

* Obligatory.

### General data of the winery

G.1 What is your annual level of red wine production? * (Mark only one).

up to 25,000 liters/year

between 25,001 and 100,000 liters/year

between 100,001 and 250,000 liters/year

between 250,001 and 500,000 liters/year more than 500,001 liters/year.

G.2 Do you have a prerequisite program in place, according to the legislation on food hygiene? * (Mark only one).

Yes/No.

G.3 Does the winery have a Critical Point Analysis System in place and Control (HACCP)? * (Mark only one).

Yes/No.

G.4 How many people work in wine production operations in the winery? *

G.5 Do winery workers have training about good manufacturing practices (GMP)? * (Mark only one).

No worker has GMP training.

More than half of workers have GMP training.

All workers have GMP training.

G.6 Do winery workers have control training and monitoring of critical control points (CCPs)? * (Mark only one).

No worker has training in control and monitoring of CCPs.

More than half of workers have training in control and monitoring of the CCPs.

All workers have training in control and monitoring of the CCPs.

### Information available on arsenic, cadmium and leads in the raw material (Critical Control Point)

ID 1. The winery has identified the legislation relating to food contamination by: (check all those you consider).

Arsenic/Cadmium/Lead.

ID 2. The winery uses the updated information available from the Spanish Agency for Food Safety and Nutrition (AESAN) on heavy metals food risk. * (Mark only one).

Yes/No.

CS 1. Does the winery have information related to physic-chemical analysis of where do the grapes used in winemaking come from? * (Mark only one).

Yes (Skip to question 11)/No.

CS 2. Does the winery have information on the fertilizers used in the fertilization of the soil from which the grapes used in winemaking come from? * (Mark only one).

Yes/No.

### Information available of the concentration of arsenic, cadmium, and lead in the soil

CS 3. The available information on the analysis of vineyard arable soil holds data on:

Select all that apply.

Total Arsenic Concentration in Soil.

Concentration of Arsenic Available in Soil.

Total Cadmium Concentration in Soil.

Cadmium Concentration Available in Soil.

Total Lead Concentration in Soil.

Lead Concentration Available in Soil.

Soil pH.

Electrical conductivity of the soil.

### Control of the raw material (analysis procedures in the winery)

CB 1. Does the winery have its own laboratory to perform chemical analysis of grapes and wines? * (Mark only one).

Yes/No.

CB 2. If you do NOT have your own laboratory, do you use an external laboratory to perform chemical analysis of grapes and wines? * (Mark only one).

Yes/No.

CB 3. Does the warehouse have the technology and personnel to perform metal analysis using atomic absorption spectrometry? * (Mark only one).

Yes/No.

CB 4. If you do NOT have your own laboratory, do you use an external laboratory to perform metal analysis using atomic absorption spectrometry on grapes and wines? *

Yes/No.

### Professional profile that performs the survey

You can tell us about your job inside the winery. (Mark only one).

Owner.

Director/Manager.

Winemaker

Winery Operator.

Administrative/management/commercial staff.

## References

[1] Lee J.C., Daraba A., Voidarou C., Rozos G., El Enshasy H.A., Varzakas T. (2021). Implementation of food safety management systems along with other management tools (HAZOP, FMEA, Ishikawa, Pareto). The case study of Listeria monocytogenes and correlation with microbiological criteria. Foods.

[2] Galani-Nikolakaki S., Kallithrakas-Kontos N., Katsanos A.A. (2002). Trace element analysis of Cretan wines and wine products. Sci. Total Environ..

[3] Vitali Čepo D., Pelajić M., Vinković Vrček I., Krivohlavek A., Žuntar I., Karoglan M. (2018). Differences in the levels of pesticides, metals, sulphites and ochratoxin A between organically and conventionally produced wines. Food Chem..

[4] Eschnauer H., Neeb R. (1988).

[5] Garcia-Sanchez A., Alvarez-Ayuso E. (2003). Arsenic in soils and waters and its relation to geology and mining activities (Salamanca Province, Spain). J. Geochem. Explor..

[6] García-Salgado S., García-Casillas D., Quijano-Nieto M.A., Bonilla-Simón M.M. (2012). Arsenic and heavy metal uptake and accumulation in native plant species from soils polluted by mining activities. Water, Air, Soil Pollut..

[7] Díez M., Simón M., Dorronsoro C., García I., Martín F. (2007). Background arsenic concentrations in Southeastern Spanish soils. Sci. Total Environ..

[8] Jiménez-Ballesta R., Bravo S., Pérez-de-los-Reyes C., Amorós J.A., García-Navarro F.J. (2023). Contents and Spatial distribution of arsenic in vineyard soils in mediterranean environment. Water, air. & Soil Pollution.

[9] Andersson A., Nilsson K.O. (1972). Enrichment of trace elements from sewage sludge fertilizer in soils and plants. Ambio.

[10] Milićević T., Aničić Urošević M., Relić D., Jovanović G., Nikolić D., Vergel K., Popović A. (2020). Environmental pollution influence to soil–plant–air system in organic vineyard: bioavailability, environmental, and health risk assessment. Environ. Sci. Pollut. Res..

[11] Codex Alimentarius Commission Codex Alimentarius (2011).

[12] World Health Organization (1998). Guidance on Regulatory Assessment of HACCP: Report of a Joint FAO/WHO Consultation on the Role of Government Agencies in Assessing HACCP.

[13] Bauman H.E. (1995). In the origin and concept of HACCP; HACCP in meat, poultry, and fish processing. Springer.

[14] Ali A.A., Fischer R.M. (2005). Implementation of HACCP to bulk cream and butter production line. Food Rev. Int..

[15] (2022). European Commission.Commission Notice (2022/C 355/01) on the implementation of food safety management systems covering Good Hygiene Practices and procedures based on the HACCP principles. including the facilitation/flexibility of the implementation in certain food businesses.

[16] European Commission (2016). Commission Notice on the implementation of food safety management systems covering prerequisite Programmes (PRPs) and procedures based on the HACCP principles, including the facilitation/flexibility of the implementation in certain food businesses. OJ C 2016.

[17] Mortimore S., Wallace C. (2013).

[18] Walker E., Pritchard C., Forsythe S. (2003). Hazard analysis critical control point and prerequisite programme implementation in small and medium size food businesses. Food Control.

[19] Sperber W.H., Pierson M.D., Corlett D.A. (1992). In Determining Critical Control Points.

[20] (2020). International Organisation of Vine and Wine Resolution OIV-OENO 630-2020. OIV Guide to Identify Hazards, Critical Control Points and Their Management in the Wine Industry.

[21] Chen H., Liu S., Chen Y., Chen C., Yang H., Chen Y. (2020). Food safety management systems based on ISO 22000: 2018 methodology of hazard analysis compared to ISO 22000: 2005. Accred Qual. Assur..

[22] Christaki T., Tzia C. (2002). Quality and safety assurance in winemaking. Food Control.

[23] Benito S. (2019). The management of compounds that influence human health in modern winemaking from an HACCP point of view. Fermentation.

[24] Martínez-Rodríguez A.J., Carrascosa A.V. (2009). HACCP to control microbial safety hazards during winemaking: ochratoxin A. Food Control.

[25] Magan N. (2006). Mycotoxin contamination of food in Europe: early detection and prevention strategies. Mycopathologia.

[26] Bermúdez Leirós V. (2020).

[27] López-Santiago J., García A.I.G., Gómez-Villarino M.T. (2022). An evaluation of food safety performance in wineries. Foods.

[28] Herce-Pagliai C., Moreno I., González G., Repetto M., Cameán A.M. (1992). Determination of total arsenic, inorganic and organic arsenic species in wine. Food Addit. Contam..

[29] (2020). OIV Collective Expertise Lead in Wine: A Review.

[30] European Commission (2021). Commission Regulation (EU) 2021/1323 of 10 August 2021 amending Regulation (EC) No 1881/2006 as regards maximum levels of cadmium in certain food stuffs. Off. J. Eur. Union L.

[31] European Commission, Commission Regulation (EC) (2006). No 1881/2006 maximum levels for certain contaminants in foodstuffs (Text with EEA relevance). Off. J. Eur. Union L.

[32] European Commission, Commission Regulation (EC) (2007). No 333/2007 of 28 March 2007 laying down the methods of sampling and analysis for the official control of the levels of lead, cadmium, mercury, inorganic tin, 3-MCPD and benzo(a)pyrene in foodstuffs (Text with EEA relevance). Off. J. Eur. Union L.

[33] European Commission (2014). Commission Recommendation of 4 April 2014 on the reduction of the presence of cadmium in foodstuffs (Text with EEA relevance). Off. J. Eur. Union L.

[34] (2015). European commission, commission recommendation (EU) 2015/1381 of 10 august 2015 on the monitoring of arsenic in food. Off. J. Eur. Union L.

[35] Organisation Internationale de la Vigne et du Vin (2022). https://www.oiv.int/standards/international-code-of-oenological-practices/annexes/maximum-acceptable-limits.

[36] Ražić Slavica, Todorović Marija, Holclajtner-Antunović Ivanka, Stoiljković Milovan (1999). Determination of metal traces in wine by argon stabilized d.c. arc. Fresenius’ J. Anal. Chem..

[37] M Mañay N., Clavijo G., Díaz L. (2009). Absorción atómica con horno de grafito. Repositorio de la Facultad de Química de la Universidad de la República, Monografía.

[38] International Organisation of Vine and Wine OIV-MA-AS323-01A Determination of arsenic in wine by atomic absorption spectrometer. https://www.oiv.int/standards/annex-a-methods-of-analysis-of-wines-and-musts/section-3-chemical-analysis/section-3-2-non-organic-compounds/section-3-2-3-other-non-organic-compounds/arsenic-%28aas%29-%28type-iv%29 (accessed 13/June/2023).

[39] (2023). International Organisation of Vine and Wine OIV-MA-AS322-10 Cadmium. https://www.oiv.int/standards/annex-a-methods-of-analysis-of-wines-and-musts/section-3-chemical-analysis/section-3-2-non-organic-compounds/section-3-2-2-cations/cadmium-%28type-iv%29.

[40] International Organisation of Vine and Wine Heavy metals by ETAAS (Type-IV) OIV-MA-F1-10 Specific methods. https://www.oiv.int/standards/compendium-of-international-methods-of-wine-and-must-analysis/annex-f/annex-f-specific-methods-for-the-analysis-of-grape-sugar/heavy-metals-by-etaas-%28type-iv%29 (accessed 13/June/2023).

[41] (2022). Federación Española del Vino El sector vitivinícola en cifras. http://www.fev.es/sector-cifras/.

[42] Ministerio de Agricultura, Pesca y Alimentación. Gobierno de España. Wines Protected Designation of Origin List. https://www.mapa.gob.es/es/alimentacion/temas/calidad-diferenciada/dop-igp/Default.aspx (accessed 28/November/2022).

[43] Asiamah N., Mensah H.K., Oteng-Abayie E. (2022). Non-probabilistic sampling in quantitative clinical research: a typology and highlights for Students and early career researchers. International Journal of Applied Research on Public Health Management.

[44] Ayhan H.Ö. (2014).

[45] Abascal E., Esteban I.G. (2005).

[46] Panisello P.J., Quantick P.C. (2001). Technical barriers to hazard analysis critical control point (HACCP). Food Control.

[47] Krishnamurti G.S.R., Naidu R. (2003). Solid–solution equilibria of cadmium in soils. Geoderma.

[48] Abascal E. (2005).

[49] Lee J.C., Neonaki M., Alexopoulos A., Varzakas T. (2023). Case studies of small-medium food enterprises around the world: major constraints and benefits from the implementation of food safety management systems. Foods.

[50] Cloquell-Ballester V., Monterde-Díaz R., Cloquell-Ballester V., Torres-Sibille A.d.C. (2008). Environmental education for small- and medium-sized enterprises: methodology and e-learning experience in the Valencian region. J. Environ. Manag..

[51] Campos L.M., de Melo Heizen D.A., Verdinelli M.A., Cauchick Miguel P.A. (2015). Environmental performance indicators: a study on ISO 14001 certified companies. J. Clean. Prod..

[52] Agencia Española de Seguridad Alimentaria y Nutrición, (AESAN), Sobre AESAN (2022), https://www.aesan.gob.es/AECOSAN/web/agencia/seccion/sobre_aesan.htm.(accessed 04/12/2022)..

[53] Agencia Española de Seguridad Alimentaria y Nutrición Metales y otros contaminantes medioambientales e industriales (2022), https://www.aesan.gob.es/AECOSAN/web/seguridad_alimentaria/ampliacion/metales_pesados.htm.(accessed 04/12/2022)..

[54] Chen C., Chiou H., Huan W., Chen S., Hsueh Y., Tseng C., Lin L., Shyu M., Lai M., Abernathy C.O., Calderon R.L., Chappell W.R. (1997). In Systemic Non-carcinogenic Effects and Developmental Toxicity of Inorganic Arsenic.

[55] Flora S.J.S., Flora G., Saxena G., Casas J.S., Sordo J. (2006). In Chapter 4 - Environmental Occurrence, Health Effects and Management of Lead Poisoning.

[56] Vela A.R., Fernández J.M. (2003). Barriers for the developing and implementation of HACCP plans: results from a Spanish regional survey. Food Control.

[57] Kourtis L.K., Arvanitoyannis I.S. (2001). Implementation of hazard analysis critical control point (HACCP) system to the alcoholic beverages industry. Food Rev. Int..

[58] Yapp C., Fairman R. (2006). Factors affecting food safety compliance within small and medium-sized enterprises: implications for regulatory and enforcement strategies. Food Control.

[59] Allam M., Bazok R., Bordewick-Dell U., Czarniecka-Skubina E., Kazimierczak R., Laikoja K., Luik A., Fuka M.M., Muleo R., Peetsmann E. (2023). Assistance needed for increasing knowledge of HACCP food safety principles for organic sector in selected EU countries. Sustainability.

[60] Yang L., Ren Q., Zheng K., Jiao Z., Ruan X., Wang Y. (2022). Migration of heavy metals in the soil-grape system and potential health risk assessment. Sci. Total Environ..

[61] Xiang M., Li Y., Yang J., Lei K., Li Y., Li F., Zheng D., Fang X., Cao Y. (2021). Heavy metal contamination risk assessment and correlation analysis of heavy metal contents in soil and crops. Environ. Pollut..

[62] Tanabe C.K., Nelson J., Ebeler S.E. (2019). Current perspective on arsenic in wines: analysis, speciation, and changes in composition during production. J. Agric. Food Chem..

